# Effect of anthropometric measurements of the hand on the development of carpal tunnel syndrome in female patients

**DOI:** 10.1097/MD.0000000000042981

**Published:** 2025-07-25

**Authors:** Memet Aslanyavrusu, Fahrettin Ege, Gülhan Sariçam

**Affiliations:** a Department of Neurology, Kayseri City Hospital, Kayseri, Turkey; b Department of Neurology, Ankara Yüksek İhtisas University, Ankara, Turkey; c Department of Neurology, Ankara Pursaklar State Hospital, Ankara, Turkey.

**Keywords:** body mass index, carpal tunnel syndrome, hand ratio

## Abstract

This study aimed to evaluate whether anthropometric measurements and body mass index (BMI) are associated with the occurrence of carpal tunnel syndrome (CTS) in female patients. This cross-sectional, observational, case-control study included 100 women with symptoms of CTS and electrophysiological evidence of median nerve entrapment at the level of the carpal tunnel. 100 volunteers without symptoms of CTS and with normal electrophysiological results were included in the study as a control group. The external dimensions of the extended hand were measured in both groups. Hand ratio (HR), wrist ratio (WR), BMI, and electrophysiological results were compared between the 2 groups. The mean age of patients with CTS was 45.5 ± 10.38 years, compared to 39.0 ± 9.96 years in the control group (*P* < .003). The BMI was higher in the CTS group (31.2 ± 4.83 kg/m²) than in the controls (25.61 ± 3.60 kg/m²; t = 8.646, *P* < .001). HR was lower in CTS patients (2.27 ± 0.11) compared to controls (2.50 ± 0.11; t = −13.881, *P* < .001), while WR was higher in the CTS group (0.74 ± 0.03 vs 0.68 ± 0.02; t = 15.905, *P* < .001). These findings suggest that women with CTS tend to have lower HRs, higher WRs, and increased BMI compared to healthy individuals. Logistic regression showed that age was not a predictor of CTS, BMI had borderline significance, and smaller HRs along with thicker wrists significantly increased CTS risk.

## 1. Introduction

Carpal tunnel syndrome (CTS) is a neuropathy of the median nerve that develops due to chronic compression of the median nerve as it passes through the carpal tunnel of the wrist.^[1]^ Ischemia and/or inflammation resulting from elevated pressure within the tunnel lead to primary segmental demyelination, which in turn causes secondary axonal damage.^[2]^ The most common clinical symptoms of CTS are pinprick and numbness in the lateral hand’s three and a half fingers *(thumb, index finger, middle finger, and half of the ring finger).* Muscle weakness is common during the late stages of nerve compression.^[3]^ CTS is the most common entrapment neuropathy, with an estimated prevalence of 3.8% in the general population. It is more prevalent in women than men.^[4]^

The following factors have been identified as potential risk factors for CTS development: female sex, diabetes mellitus, family history, age, and wrist trauma.^[5]^ The elevated prevalence in pregnant and lactating women indicates that hormonal disparities may contribute to the observed variability in CTS.^[6,7]^ Furthermore, numerous studies have linked elevated body mass index (BMI) to a higher prevalence of CTS. This association may be attributed to increased adipose tissue in the wrist, which reduces the area available for the median nerve beneath the flexor retinaculum.^[8]^ Some patients diagnosed with CTS are susceptible to repetitive and forceful movements despite the absence of any identifiable risk factors.^[9]^

Chiotis et al investigated the relationship between CTS and anthropomorphic measurements of the hand.^[10]^ Several other studies have questioned these relationships, and the connection between CTS and changes in hand morphology has been revealed.^[11,12]^ Another study investigated the relationship between metacarpal indices calculated by posterior-anterior radiographic measurements and CTS.^[13]^

This study aimed to investigate the relationship between anthropomorphic measurements, BMI, and disease in the female population, where CTS is more common, and to reveal whether hand morphology, especially in women, creates a predisposition to the disease.

## 2. Methods

### 2.1. Study design and participants

This cross-sectional, observational, case-control study was conducted at our neurology clinic between March and July 2024. The study included 100 women diagnosed with CTS based on clinical symptoms and electrophysiological confirmation of median nerve entrapment, and 100 healthy women as the control group. The patient group consisted of individuals with symptoms of CTS *(paresthesia, nocturnal pain in the median nerve trace area, etc*) and electrophysiological evidence of entrapment at the carpal tunnel level. The control group did not report CTS symptoms and had normal median nerve electrophysiological parameters. The control group was selected from outpatients who were as close as possible age to the patients diagnosed with CTS. The manuscript is compatible with *strengthening the reporting of observational studies in epidemiology* guidelines.

Pregnant women, lactating women, patients with diabetes mellitus, polyneuropathy of any etiology, rheumatological diseases, hypothyroidism, and traumatic nerve injury in the upper extremities were excluded.

All procedures followed were in accordance with the ethical standards of the responsible committee on human experimentation *(institutional and national*) and with the Helsinki Declaration of 1975, as revised in 2008. Our institution has granted ethics committee approval with protocol number *24-81*, and informed consent has been obtained from all participants.

Although nonparametric tests were primarily used in our study due to non-normal data distribution, the post hoc power analysis was performed based on the wrist ratio (WR) comparison using an independent samples *t*-test in G*Power 3.1, in line with its software requirements. An effect size of *R* = 0.8247 was calculated, with an assumed alpha level of 0.05. Based on the sample size (100 patients and 100 controls), the power of the study was found to be 1.00 (100%).

### 2.2. Anthropometric measurements

**Figure 1.**
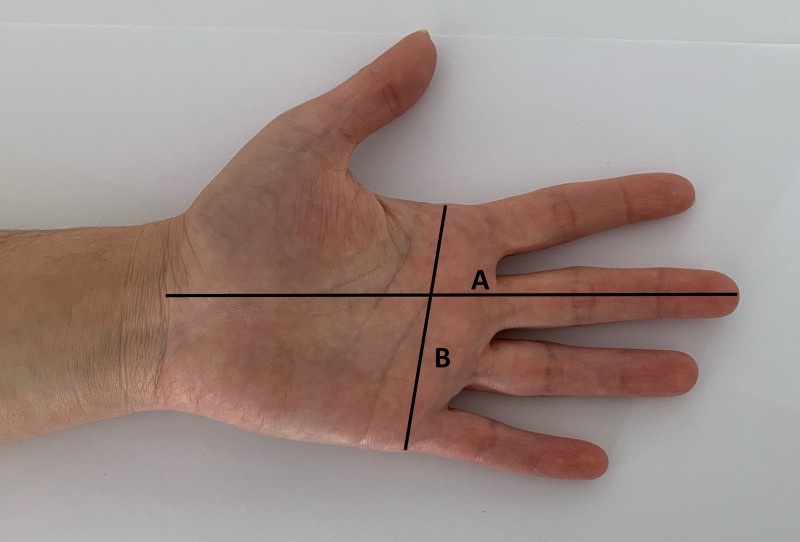
Measurement method for calculating hand ratio.

**Figure 2.**
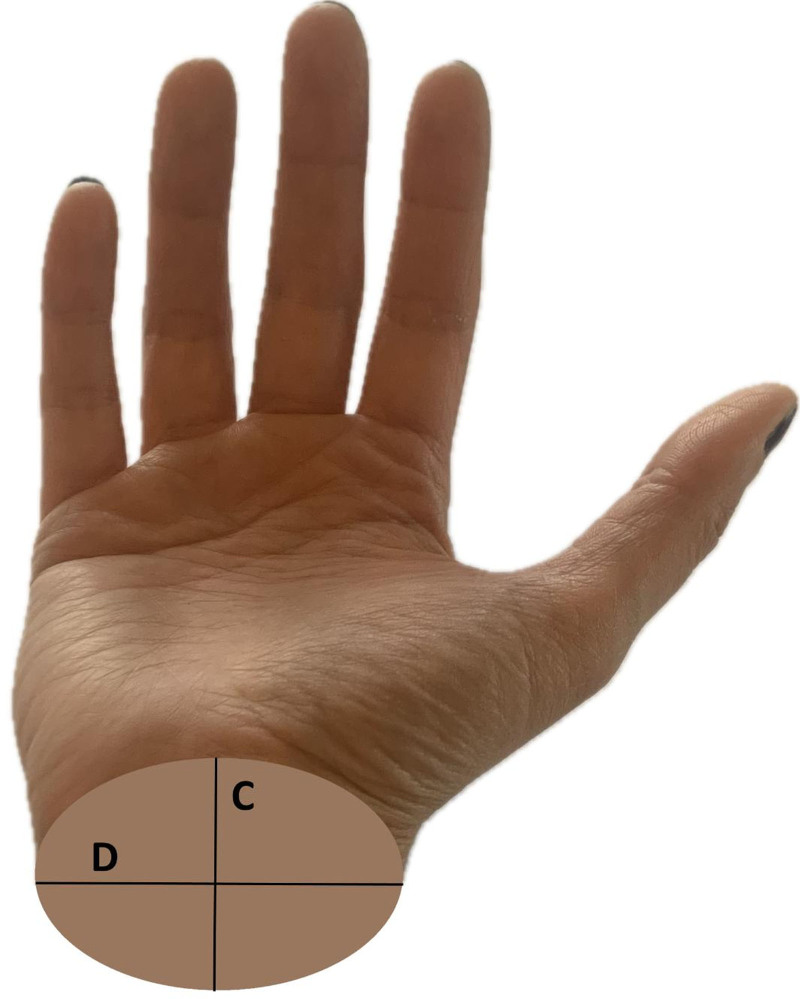
Measurement method for calculating wrist ratio.

The height and weight of all patients participating in the study were measured, and their BMI was calculated. In patients with bilateral CTS, anthropometric measurements were taken from the hand with greater CTS. In patients with bilateral CTS, measurements were taken from the more severely affected hand. If both hands were equally affected, measurements were taken from the dominant hand. Hand anthropometric measurements were obtained using a digital caliper (Insize 1108-150, Insize Co., China). Anthropometric measurements of hand length, hand width, wrist depth, and wrist width were performed in accordance with the method described by Chiotis et al, with the participants seated, forearm supinated, and fingers fully extended.^[10]^ The distance between the distal part of the third finger and the distal flexor fold on the volar aspect of the wrist was used to measure the hand length. The palm’s width was determined by measuring the most significant distance between the metacarpal heads of the index and fifth fingers. The hand ratio (HR) was calculated by dividing the hand length by the hand width (Fig. 1). The external dimensions of the wrist were measured at the level of the distal flexor crease, including the palmo-dorsal *(depth*) and mediolateral *(width*) dimensions. The WR was calculated as the depth divided by width (Fig. 2).

### 2.3. Electrophysiology

Nerve conduction studies (NCSs) were performed according to the guidelines of the American Association of Neuromuscular and Electrodiagnostic Medicine recommended for CTS. Median nerve sensory conduction was evaluated using a 13 to 14 cm conduction distance over the wrist. Median sensory conduction over the wrist was compared with ulnar sensory conduction in the same extremity. Median nerve motor conduction was recorded from the thenar muscle. Median motor nerve distal latency was compared with ulnar motor nerve distal latency. Median and ulnar nerve’s fourth finger sensory conduction response peak latencies were compared.^[14]^ Normal threshold values were determined based on values suggested by Shapiro and Preston.^[15]^

The electrophysiology laboratory was kept at 22°C to 23°C, and the skin temperature was maintained above 32°C during the measurements. NCSs were conducted using the *Dantec Keypoint G4, EMG/NCS/EP device (Natus Neurology, Denmark*). Motor and sensory conduction of the median and ulnar nerves of both hands were studied in both groups. A motor distal latency above 3.8 ms and/or antidromic sensory nerve conduction velocity calculated according to the onset latency below 49 m/s were considered indicative of CTS. To exclude polyneuropathy in the patient group, all participants’ bilateral common peroneal and posterior tibial nerve motor and sural nerve sensory conduction were also measured, and people with abnormal results were excluded from the study at this stage. In the healthy group, measurements were obtained from the dominant hand. All measurements were performed by a single neurologist experienced in electrophysiology, in accordance with the American Association of Neuromuscular and Electrodiagnostic Medicine guidelines.^[14]^

### 2.4. Statistical analysis

The patient data collected within the scope of the study were analyzed using the IBM Statistical Package for the Social Sciences for Windows 29.0 (IBM Corp., Armonk). Frequency and percentage were given for categorical data, while mean and standard deviation were provided as descriptive values for continuous data. The Kolmogorov–Smirnov test revealed that the groups did not follow a normal distribution.^[16]^ Therefore, nonparametric tests were preferred instead of parametric tests. The Mann–Whitney *U* test was used to compare age, BMI, HR, and WR values. Logistic regression analysis was conducted to determine whether BMI, HR, and WR were independent predictors. Additionally, receiver operating characteristic analysis was performed to determine the cutoff values for HR and WR. Results were considered statistically significant when the *P*-value was <.05.

## 3. Results

The mean age of patients with CTS was 45.5 ± 10.38 years, which was significantly higher than that of the control group (*39 ± 9.96 years; P < .003*). The BMI was also significantly higher in the CTS group (31.2 ± 4.83 kg/m^2^) compared to the healthy controls (*25.61 ± 3.60 kg/m*^*2*^*; t = 8.646, P < .001*). The HR was significantly lower in CTS patients (*2.27 ± 0.11*) than in the control group (*2.50 ± 0.11; t = −13.881, P < .001*), while the WR was significantly higher in CTS patients (*0.74 ± 0.03*) compared to controls (*0.68 ± 0.02; t = 15.905, P < .001*). These findings indicate that individuals with CTS tend to have smaller HRs but higher BMI and WRs than healthy individuals (Table 1).

**Table 1:** Anthropometric measurements of CTS patients and control group.

	CTS (n = 100) Medyan ± SD	Controls (n = 100) Medyan ± SD	Mann–Whitney *U*	Z	*P*-value
Age	45.5 ± 10.38	39 ± 9.96	3774.0	−2.997	<.003*
BMI (kg/m2)	31.2 ± 4.83	25.61 ± 3.60	2000.0	−7.33	<.001*
HR	2.27 ± 0.11	2.50 ± 0.11	876.5	−10.079	<.001*
WR	0.74 ± 0.03	0.68 ± 0.02	541.5	−10.928	<.001*

BMI = body mass index, CTS = carpal tunnel syndrome, HR = hand ratio, SD = standard deviation, WR = wrist ratio.

* *P* < .01, Mann–Whitney *U*.

A logistic regression analysis was performed to determine the power of variables in predicting CTS. According to these results, it was determined that age did not have an independent effect on CTS (*P = .864*); *BMI* may have a slight borderline effect (*P = .057*). The risk of CTS decreases significantly as the HR increases (*P < .001*). Small hands significantly increase the risk of CTS. The risk of CTS increases considerably as the WR increases (*P < .001*). We found that the probability of developing CTS is higher in individuals with thicker wrists (Table 2).

**Table 2:** Logistic regression analysis results.

	*B*	Wald	Odds ratio (Exp(B))	95% CI	*P*-value
Age	0.005	0.02	0.501	−0.05–0.06	.864
BMI (kg/m^2^)	0.144	3.61	0.536	−0.04–0.29	.057
HR	−13.122	17.27	2.001	−19.31–6.93	<.001
WR	96.264	21.62	1.0	55.69–136.83	<.001

BMI = body mass index, HR = hand ratio, WR = wrist ratio.

**Figure 3.**
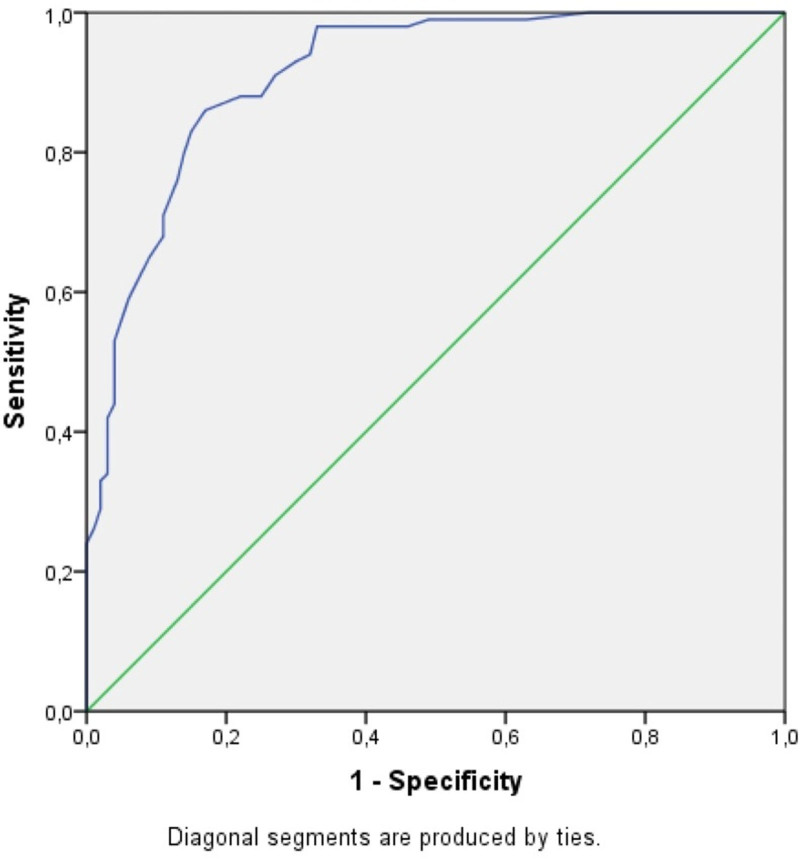
ROC curve for hand ratio. ROC = receiver operating characteristic.

**Figure 4.**
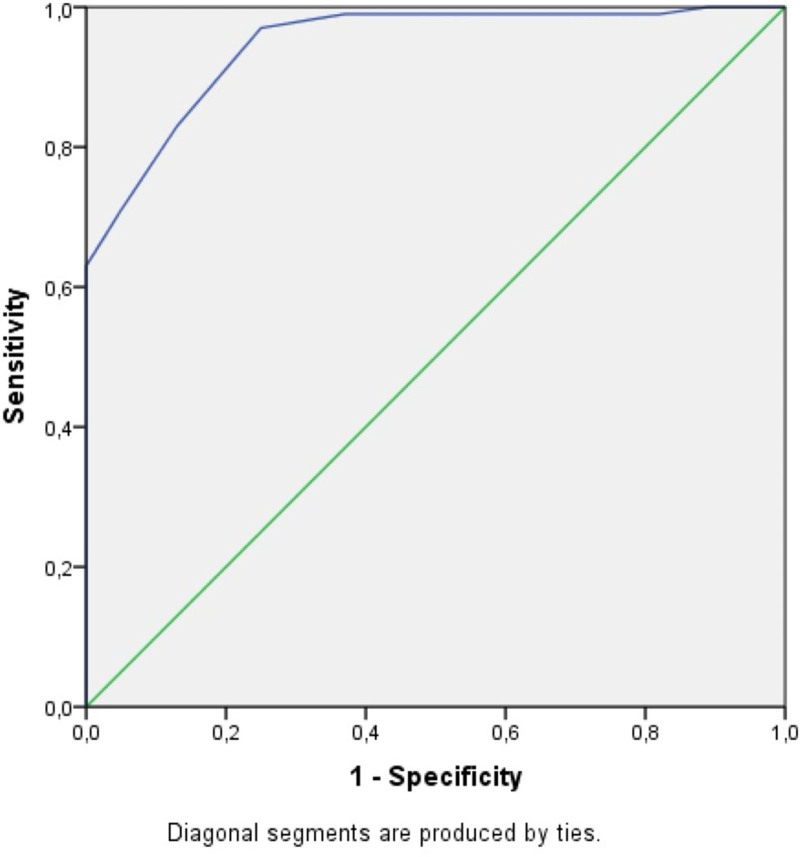
ROC curve for wrist ratio. ROC = receiver operating characteristic.

Receiver operating characteristic curve analysis and Youden Index method were used to determine the optimal cutoff values for HR and WR in diagnosing CTS. For the HR, the optimal cutoff was 2.37, with a sensitivity of 86% and specificity of 83%. For the WR, the cutoff was 0.69, yielding a sensitivity of 97% and specificity of 75%. These findings indicate that while both HR and WR are useful diagnostic parameters, WR demonstrates higher sensitivity and may serve as a more effective indicator for CTS (Figs. 3 and 4).

## 4. Discussion

Our results demonstrated that the BMI was significantly higher in patients with CTS. Furthermore, the HR was smaller in patients with CTS than in healthy individuals. This suggests that lower HRs (shorter hand length relative to width) are associated with a higher likelihood of CTS in these female patients. In contrast, median nerve damage was more prevalent in patients with higher WR. This means entrapment was more common in wrists with greater thickness, width, and a square shape.

The following risk factors have been identified to contribute to the development of CTS: age, sex, diabetes mellitus, hypothyroidism, obesity, tobacco use, hand injury, pregnancy, lactation, occupational risk factors, repetitive wrist movements, and repetitive microtrauma.^[6,7]^ Nevertheless, a specific etiological factor may not be identified in approximately 50% of CTS patients.^[17]^ These idiopathic forms may be related to sex and individual variations, such as the hand/wrist structure and shape.

Our findings are consistent with those of Chiotis et al,^[10]^ who also reported that individuals with lower HRs and higher WRs had a greater risk of developing CTS. Similarly, Trybus et al^[12]^ observed this specific hand morphology – characterized by a broader and shorter hand structure – in a cohort of 48 CTS patients, supporting the idea that certain anthropometric patterns may predispose individuals to median nerve compression. Mokhtarinia et al^[11]^ also identified higher WR values in CTS patients but did not find BMI to be an independent risk factor. In contrast, our results suggest that BMI may still play a contributory role in CTS development, in addition to hand and wrist morphology. Although Sahabelam et al^[13]^ demonstrated an association between hand morphology and CTS using radiographic analysis, methodological differences and the use of distinct measurement indices limit the comparability of their findings with ours.

Previous studies have demonstrated a correlation between an increase in BMI, an increase in motor distal latency, and a slowing of sensory conduction velocity in patients with CTS. It has been demonstrated that the extent of myelin sheath damage gradually increases with increasing BMI.^[18,19]^ It has also been postulated that the accumulation of adipose tissue within the carpal canal may result in compression of the median nerve within the carpal tunnel, thereby inducing ischemia and myelin sheath damage near the node of Ranvier.^[20]^ While low body weight is considered a protective factor, being overweight and obese are risk factors for CTS. Furthermore, BMI may be an essential indicator of CTS severity.^[21]^ However, these studies found no correlation between BMI and hand/wrist structure and shape.

In our study, we similarly found a significantly higher BMI in patients with CTS, supporting the hypothesis that increased body mass may contribute to the development of median nerve entrapment. However, we did not assess CTS severity or electrophysiological parameters, which limits direct comparison with these prior findings.

The idiopathic form of CTS has been associated with excessive or repetitive hand use. External hand dimensions are considered valuable for assessing the predisposition to develop CTS in individuals exposed to repetitive manual activities.^[22,23]^ Supporting this view, Chiotis et al^[10]^ demonstrated a correlation between external hand measurements and ultrasonographic findings of the carpal tunnel, showing that the carpal tunnel tended to be square in shape and that ultrasonographic estimates were not superior to external measurements in identifying individuals at risk. Our study supports this notion by demonstrating that specific external anthropometric features – particularly a higher WR – were significantly associated with CTS. These findings suggest that simple hand and wrist measurements may be sufficient for identifying individuals at increased risk, especially in settings where imaging is not readily available.

The higher prevalence of CTS among women remains incompletely understood. One possible explanation is the greater propensity for weight gain in women, particularly during the postmenopausal period, which may contribute to increased compression within the carpal tunnel.^[24]^ Estrogen receptors are present in peripheral nerves and play a role in neuronal proliferation and regeneration. Declining estrogen levels after menopause may impair the regenerative capacity of the median nerve, potentially increasing susceptibility to CTS. Supporting this hypothesis, Al-Rousan et al^[25]^ reported that hormone replacement therapy might have a protective effect against CTS in postmenopausal women aged 65 years and older. Additionally, repetitive hand use related to domestic responsibilities, such as housework and childcare, has been associated with increased CTS incidence in women.^[26]^ Considering these sex-related biological and behavioral differences, we limited our study population to women in order to maintain a homogeneous sample and to better evaluate the relationship between anthropometric factors and CTS risk in this higher-risk group.

This study had some limitations that may restrict the generalizability of the findings. It was a single-center experience, and all participants were female, which, while providing a homogeneous sample, limits applicability to the broader population. CTS was not divided into mild, moderate, and severe categories, so the relationship between HR and WR values and disease severity could not be evaluated. Moreover, no ultrasonographic assessment was performed, preventing comparison between external anthropometric measures and internal structural features of the carpal tunnel. Additionally, potential confounding variables, such as occupational hand use and repetitive daily wrist activity, were not analyzed.

Future studies should aim to validate the diagnostic value of HR and WR in more diverse populations, stratify participants by CTS severity, and incorporate imaging and electrophysiological data to better understand how anatomical and metabolic factors jointly influence the development and progression of CTS.

## 5. Conclusion

Our findings indicate that smaller HRs and larger WRs are significantly associated with CTS, whereas age was not found to be a contributing factor. BMI showed a weak association. Hand and wrist measurements may be valuable in identifying individuals at risk for CTS.

Supplemental Digital Content is available for this article (https://links.lww.com/MD/P469).

## Author contributions

**Conceptualization:** Fahrettin Ege.

**Data curation:** Memet Aslanyavrusu, Gülhan Sariçam.

**Methodology:** Memet Aslanyavrusu, Gülhan Sariçam.

**Software:** Memet Aslanyavrusu, Fahrettin Ege.

**Visualization:** Gülhan Sariçam.

**Writing – original draft:** Memet Aslanyavrusu.

**Writing – review & editing:** Memet Aslanyavrusu, Fahrettin Ege, Gülhan Sariçam.

## Supplementary Material
