# Supplementary material for: Effect of anthropometric measurements of the hand on the development of carpal tunnel syndrome in female patients
Source: Medicine (Baltimore). 2025 Jul 25;104(30):e42981. doi: 10.1097/MD.0000000000042981 (PMC12303490; doi:10.1097/MD.0000000000042981)
Supplement: Supplementary file 1 [file medi-104-e42981-s001.docx]

Assessed Foreligibility (n=300)

Excluded (n=100)

Not meeting inclusion criteria (n=80)

Declined to participate (n=8)

Other reasons (n=12)

200 patients were included

Patient group (n=100) Control group (n=100)

CTS symptoms and positive No CTS symptoms, normal

electrophysiological result electrophysiological result
